# Reference data on anthropometrics, aerobic fitness and muscle strength in young Norwegian men and women

**DOI:** 10.1007/s00421-021-04784-4

**Published:** 2021-08-14

**Authors:** Anders Aandstad

**Affiliations:** Section for Military Leadership and Sport, Norwegian Defence University College, P.O. Box 1550 Sentrum, N-0015 Oslo, Norway

**Keywords:** Normative, BMI, Maximal oxygen uptake, Cardiorespiratory, Military

## Abstract

**Purpose:**

Anthropometrics, aerobic fitness and muscle strength are measured in one-third of all 18-year-old Norwegian men and women during yearly selection for compulsory military service. The large sample size and geographical representativity make these data valuable for reference. The main purpose of this study was to present reference data for anthropometrics and physical fitness in young Norwegian men and women.

**Methods:**

All 154,659 subjects (66% men and 34% women, 17–21 years old) who completed physical examinations at conscript selection from 2011 to 2019 were included in the study. Body mass index (BMI) was calculated from height and weight measurements. Peak oxygen uptake (VO_2peak_) was estimated from performance on a maximal treadmill test. Muscle strength was measured by isometric chest and leg press, or seated medicine ball throw, standing long jump and pull-ups.

**Results:**

Mean BMI (SD) was 23.1 (3.4) and 22.9 (3.3) kg·m^−2^ in men and women, respectively (*P* < 0.001), and 24% of men and 21% of women had a BMI ≥ 25 kg·m^−2^. Estimated VO_2peak_ was 52.9 (4.6) and 42.7 (3.9) mL·kg^−1^·min^−1^ in men and women, respectively (*P* < 0.001). Men performed significantly better than women on all muscle strength tests, with corresponding effect sizes varying from 1.14 for isometric leg press to 2.96 for seated medicine ball throw.

**Conclusion:**

The presented reference data on physical fitness in young Norwegian men and women can be used to evaluate population health, serve as reference material for future studies and describes sex differences in several physical fitness parameters.

**Supplementary Information:**

The online version contains supplementary material available at 10.1007/s00421-021-04784-4.

## Introduction

It is well established that aerobic fitness and anthropometrics are linked to cardiovascular disease risk and all-cause mortality (Mitchell et al. 2010), and there is also evidence for several health benefits related to resistance training and muscle strength (Liu et al. 2019). Descriptive studies on physical fitness may, therefore, contribute to evaluation of health status in populations and serve as reference for future studies investigating secular changes in health-related physical fitness.

Reference data on physical fitness can be collected from self-reported fitness or objective measurements. The latter is usually considered superior due to higher validity (Obling et al. 2015). Yet, objective measurements are time-consuming, labor-intensive, and costly. This typically leads to reduced sample sizes and geographical catchment areas. Many reference studies on physical fitness are also hampered by relatively low participation rate and possible bias caused by self-selection (Loe et al. 2014; Aadland et al. 2017).

The abovementioned challenges may be less prominent if studying civilians during selection for obligatory military service. Norway practices conscription for both genders, and a two-step system is used to select young men and women into service (Teien et al. 2019). In step one, all Norwegian 17-year-old men and women (*n* ≈ 60,000) are required to complete a 55-item internet questionnaire related to their health, motivation for military service, education background, and physical fitness level. The adherence rate to this questionnaire is ≥ 95%. Based on the answers in step one, one-third of the population (*n* ≈ 20,000, 30–40% women) is annually selected to participate in step two of the selection process. This includes a 1-day visit to a conscript selection center and is usually carried out when the subjects are 18 years old. Here, the candidates undergo a medical check, height and weight measurements, psychological tests and objective measurements of aerobic fitness and muscle strength. Ultimately, approximately 9000 subjects (33% women in 2020) are annually selected to conduct the 1-year long military service (Norwegian Armed Forces 2020).

Objective physical fitness testing at Norwegian conscript selection was re-introduced in 2011 and data on physical fitness are, therefore, available for ~ 150,000 civilian young men and women for the 2011–2019 period. No previous reference studies on physical fitness in Norway are based on such a large sample size and with similar nationwide geographical representativity (Haugen et al. 2014; Kjær et al. 2016; Kolle et al. 2010; Loe et al. 2014; Aadland et al. 2017). In other countries, large-scale studies on fitness have been published from conscript selection in Sweden and Switzerland (Henriksson et al. 2020; Wyss et al. 2019), and from conscript service in Finland (Santtila et al. 2018). However, none of these three studies included women, and the Swedish and Finnish studies had other aims than presenting reference values for physical fitness.

One limitation exists for reference data collected during Norwegian conscript selection: relatively few of the subjects who report low fitness level during step one will be selected for participation in step two. This will introduce bias into the objective step two fitness data when compared to the general population. However, the amount of bias can possibly be estimated, since some subjects with low self-reported fitness are still included in the step two examinations, and step one data on self-reported fitness are available for almost the entire population. Such missing data analyses are usually not possible in other reference studies.

While reference data are important when evaluating population health, such data may also play an important role in occupational, school or sport settings. For instance, norms for physical fitness can be beneficial when evaluating students in physical education classes or when establishing cutoff values for acceptancy into physically demanding occupations. In such situations, it is often necessary to differentiate the scales and requirements by sex. Sex differences in physical performance vary greatly according to which fitness component is measured (Bishop et al. 1987; Kjær et al. 2016). Thus, conscript selection data can give valuable information about sex differences in aerobic fitness and muscle strength and power measured from several tests. A better understanding of the sex differences in physical performance will be beneficial when targeting training for both men and women.

Accordingly, the primary aim of the current study was to present reference values for anthropometrics, aerobic fitness and muscle strength and power in a large sample of healthy young Norwegian men and women. Secondarily, the reference data will be used to investigate sex differences in several physical fitness measurements.

## Materials and methods

This study can be characterized as a descriptive cross-sectional study. It was approved by the Research Group at the Norwegian Defense University College, while the Norwegian Centre for Research Data and the Regional Committee for Medical and Health Research Ethics considered the study to be exempted from notification (due to use of anonymous register data only). The data were extracted from the database P3 by technical personnel from the Norwegian Armed Forces HR and Conscription Centre (Hamar, Norway).

### Subjects

All men and women who performed step two examinations at one of the Norwegian military conscription centers between autumn 2011 and spring 2019 were included in the study. An exception was for subjects ≥ 22 years old (1.3% of the initial sample size), who were removed from the data set, as they were considered atypical for the population. Altogether 154,659 subjects were included in the analysis, with 66.4% being men (*n* = 102,702) and 33.6% women (*n* = 51,957). Age ranged from 17 to 21 years, with mean (SD) age of 18.8 (0.8) and 18.6 (0.6) years in men and women, respectively. The included subjects were recruited from all 19 counties in Norway (K. Olsen, Norwegian Armed Forces HR and Conscription Centre, 2021, pers. comm.), but information was not available regarding county for each subject.

**Fig. 1:**
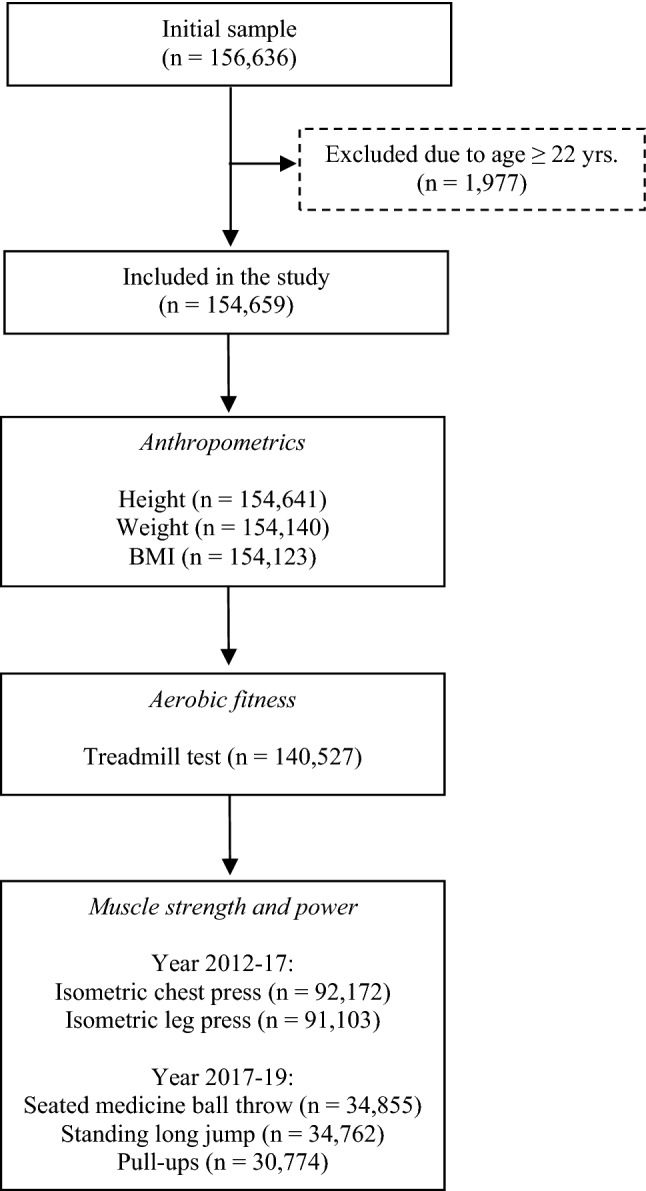
Flowchart of participation and measurements

Reported sample size varied among the different measurement variables (Fig. 1). Reasons for missing data pertaining individual subjects and measurements were not available. However, some subjects were dismissed after failing the medical screening, and therefore, only carried out weight and height measurements. Other subjects did not participate in all tests for medical reasons (injuries, etc.) or because of temporarily faulty test equipment. Muscle strength tests were implemented 1 year after the aerobic fitness test; thus, fewer subjects completed muscle strength measurements. In addition, two different strength test batteries were used over the course of the data collection period, i.e., no subjects have performed all described muscle strength tests. Finally, some conscription centers lacked pull-ups equipment during the first months of test implementation, which led to a reduced sample size for this test. Data for at least one anthropometrical or physical fitness measurement had to be registered to be included in the study.

### Measurements

Military medical doctors carried out the weight and height measurements, while military selection officers acted as test leaders for the physical fitness measurements. The subjects performed the running test and the muscle strength and power tests dressed in running shoes and sports attire.

The same aerobic fitness test was administered for the entire data collection period from fall 2011 to spring 2019 (8 years). The isometric chest and leg press were administered between fall 2012 and spring 2017 (5 years) but were replaced by a strength test battery consisting of seated medicine ball throw, standing long jump and pull-ups between fall 2017 and spring 2019 (2 years).

At all conscript selection centers, the physical fitness tests were administered according to official test regulations (Frantzen 2020; The Norwegian Armed Forces 2012; Aandstad 2017).

#### Anthropometrics

Height (to the nearest cm) and weight (to the nearest kg) were measured with a wall-mounted stadiometer and a mechanical or digital weight scale, respectively. Body mass index (BMI) was calculated by dividing weight in kg by height in m squared (kg·m^−2^). Individual BMI values were classified according to established cutoff values for underweight (< 18.5 kg·m^−2^), normal weight (18.5–24.9 kg·m^−2^), overweight (25.0–29.9 kg·m^−2^) and obesity (≥ 30.0 kg·m^−2^) (World Health Organization 2000).

#### Aerobic fitness

A maximal treadmill test was used to evaluate aerobic fitness. Details of this test protocol are presented in an earlier publication (Aandstad and Hageberg 2019). In short, the test began with 6 min of walking at 5 and 10% incline. Thereafter, the treadmill speed was automatically increased by 1 km·h^−1^ every minute (10% incline) until voluntarily exhaustion. Run time in minutes and seconds was registered to the nearest 5 s. The test protocol was thoroughly explained and demonstrated for the subjects before start. Each conscription center had access to five or ten treadmills which were placed side by side. Accordingly, up to ten subjects were tested simultaneously. The same type of treadmill (T300, Nordic Sportsmaster AS, Nesbru, Norway) was used at all conscription centers for the entire test period. The treadmills were calibrated for inclination and speed once a month.

Peak oxygen uptake was estimated from run time and sex based on the following prediction equation:

*Ŷ* = 19.8 + 0.047run time (seconds) − 4.6sex (men = 0, women = 1).

Reliability and validity of the treadmill test and the prediction equation have previously been reported for male and female conscript soldiers (Aandstad and Hageberg 2019). Test–retest analyses of run time produced an intraclass correlation coefficient (ICC) of 0.95 (0.91, 0.97), while 95% limits of agreement was ± 60 s. The validity analyses demonstrated a Pearson correlation coefficient (*r*) of 0.89 (0.83, 0.93) between estimated and directly measured maximal oxygen uptake, and 95% limits of agreement of ± 5.6 mL·kg^−1^·min^−1^.

#### Muscle strength and power

Isometric chest and leg press were performed in a custom-made apparatus (Norwegian Defence Logistics Organization, Horten, Norway) and according to official test regulations (The Norwegian Armed Forces 2012). Illustration of the apparatus is given in Online Resource 1. The test leader first explained and demonstrated the two tests. Thereafter, the subjects carried out a short warm-up procedure consisting of 2 × 5 push-ups and 2 × 5 unloaded deep squats, including light stretching.

The isometric chest press was performed seated in the apparatus and with the back against the backrest. The subject grasped the hand-bar and the test leader adjusted the position of the seat and the height of the bar so that the bar was in front of the chest and the elbows were at 110° angles. The subject then pushed the bar with maximal effort for 5 s. Peak force was registered by a load cell connected to the bar, and the result was registered to the nearest 1 kg. Another attempt was performed after a 30 s break. If the second attempt produced a result > 10% better than the first, a third attempt was given after an additional 30 s break. The best result of the 2 to 3 attempts was registered.

For the isometric leg press, the subject remained seated in the apparatus, grasping the handles on the sides of the seat. The subject placed his or her feet on the leg-bar and the test leader adjusted the position of the seat so that the knees were bent at 120° angles. The subject then pressed on the bar (connected to a load cell) with maximal effort for 5 s. The number of attempts, length of breaks and data registration were identical to the chest press exercise. A potential maximum result was restricted to 500 kg for both the chest and leg press.

The seated medicine ball throw, standing long jump and pull-ups were performed according to official test regulations (Frantzen 2020; Aandstad 2017), see also illustrations presented in Online Resource 2–5. The test leader first demonstrated and explained the events. The subjects then performed a short warm-up procedure consisting of 1 min jogging in place, 1 × 10 push-ups, 1 × 10 unloaded deep squats, 2 × 5 unloaded split squats (5 on each leg), 5 vertical and 2 horizontal jumps (submaximal effort), and light stretching.

The seated medicine ball throw was performed in a customized weight bench (Gym 2000, Vikersund, Norway). The bench settings were similar for all subjects tested. Starting position was with the subject seated in the bench holding a 10 kg medicine ball (Trial SRL, Forli, Italy) to the chest. The medicine ball was then pushed with maximal power as far as possible. The subject was instructed to always maintain contact between the back rest of the bench and the subject’s back and head. The length of the throw was measured from localizing the center of the ball’s impact point to the nearest 10 cm by use of a customized measurement mat. The best result of two attempts was recorded.

The standing long jump was performed with the subject standing behind a line on the measurement mat. The ankles and knees were flexed, and arms were swung to enhance the forward propelling movement of the body in an attempt to jump as far as possible. The landing spot for the most rear part of the shoes (or body) was identified, and the jump was measured to the nearest 5 cm. It was not necessary to stand still after landing on the mat. The best result of two attempts was recorded.

The starting position for pull-ups was hanging vertically from a convex-shaped 2 × 6ʺ beam using an overhand grasp and with straight arms and legs. The subject then raised the body until the chin was over the beam, followed by lowering the body until the arms were fully stretched. An accepted attempt was performed in a smooth and controlled manner; kicking or swinging the body was not allowed. The total number of accepted repetitions was registered. If a subject was not able to perform any vertical pull-ups, an alternative horizontal pull-ups test was administered. Here, the starting position was with the subject grasping the beam with an overhand grip (straight arms) and with heels placed on a bench to achieve a horizontal starting position. The straight body was raised until the chest touched the underside of the beam. Again, only smooth and controlled movements were accepted and swinging the body was not allowed. The total number of accepted repetitions was registered.

Validity of all five muscle strength tests is previously investigated in 40 young men and women during conscript selection step two (Aandstad 2015). All tests correlated significantly (*r* = 0.61–0.86) with a timed casualty evacuation test (pulling a 70 kg manikin 35 m) and with 1 repetition maximum bench press performance (*r* = 0.68–0.93). Reliability of the medicine ball throw, standing long jump and pull-ups were investigated in 33 male and female conscript soldiers (Aandstad and Kirknes 2018). The study demonstrated test–retest ICC to be 0.95–0.96 in all three tests. No published data exist for reliability of the two isometric tests.

### Supplementary study

The step one selection introduces a possible bias towards more high fit individuals participating in the step two physical fitness examinations. Thus, some additional data were analyzed to try to quantify the magnitude of this selection bias. All self-reported data on anthropometrics and physical fitness from step one of the conscript selection were available for men and women born in 1996 (*n* = 61,086) and in 2000 (*n* = 57,644). Approximately, one-third of these subjects also completed the step two examinations. Accordingly, the relationship between self-reported (step one) and objective (step two) data were used to produce adjusted mean values for all anthropometrical and physical fitness variables. A more comprehensive description of this supplementary study is given in Online Resource 7.

### Statistical analysis

All outcome variables were checked for normality by visual inspections of data distribution plots (histograms). All data were treated as normally distributed, except pull-ups performance which was positively skewed. Pull-ups also produced a non-normal distribution because of the two-tier protocol. Data points considered to be mistyped in the P3 register (unnatural outliers) were removed prior to conducting analyses. For each variable, no more than 0.06% (*n* ≤ 36) of the data points were excluded for this reason.

Descriptive data are presented as mean ± standard deviation (SD), except for pull-ups which are presented as median including 25–75 percentiles. An independent sample’s *t* test was used to check for significant differences between sexes for all variables, except for the pull-ups variable for which the Mann–Whitney *U* test was utilized. Mean differences between sexes are presented with 95% confidence intervals (CI). Effect sizes were calculated as Cohen’s *d*. The distribution of scores for the various measurements is presented graphically as cumulative relative frequency, as well as percentile values. The Chi-square test was used to examine differences in frequencies for BMI categories between men and women and followed up by pairwise comparisons of expected and observed frequencies by use of a binominal test.

Statistical analyses were performed in jamovi (version 1.2.27; The jamovi project, Sydney, Australia) and GraphPad (version 8.4.3; GraphPad Software, San Diego, California, USA). The latter was only used for the Chi-square testing and to create the frequency graphs. A probability (*P*) of < 0.05 was considered statistically significant.

## Results

**Fig. 2:**
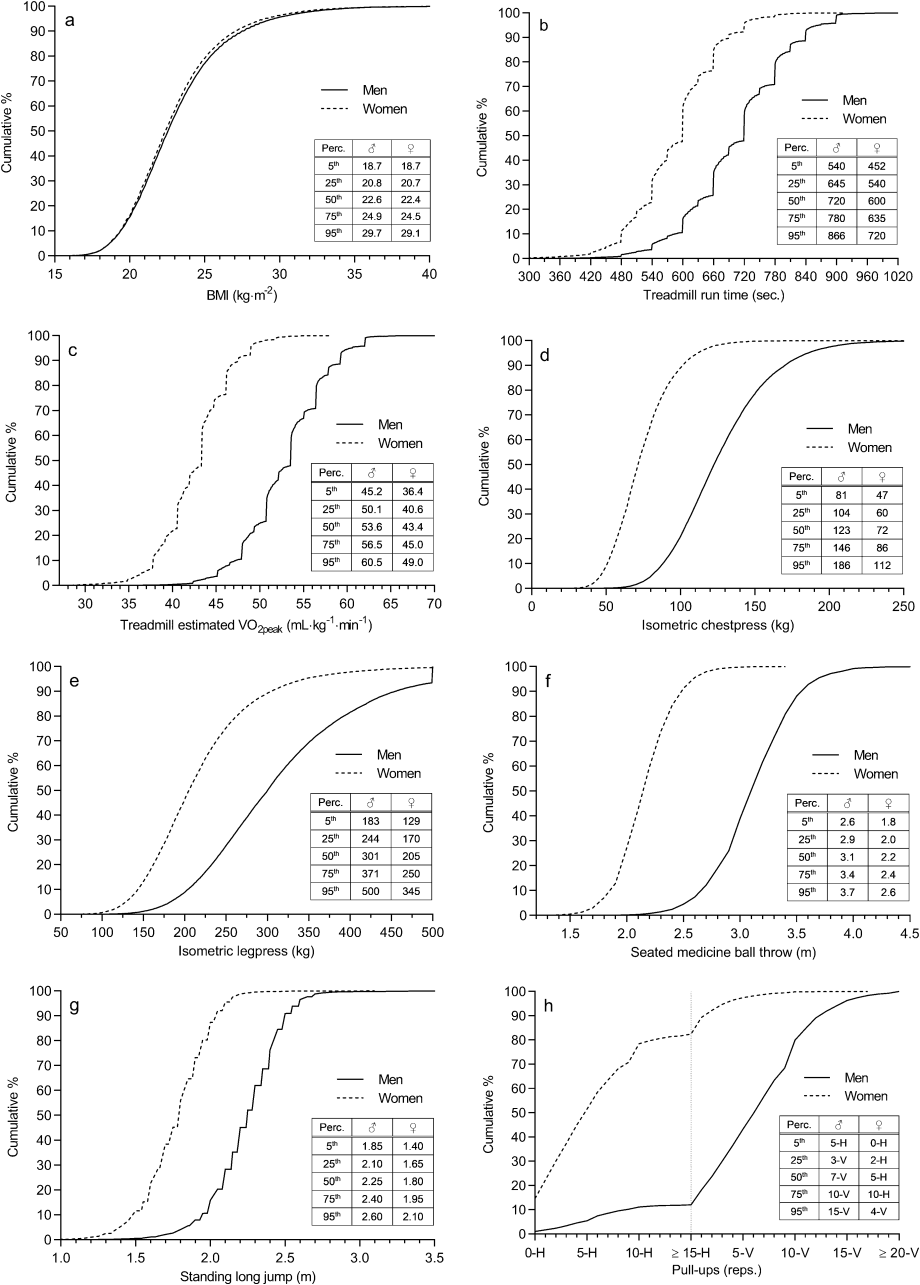
Cumulative relative frequency and selected percentile (perc.) values for body mass index (panel **a**), treadmill run time (panel **b**), treadmill estimated peak oxygen uptake (panel **c**), isometric chest press (panel **d**), isometric leg press (panel e), seated medicine ball throw (panel **f**), standing long jump (panel **g**) and vertical (V) or horizontal (H) pull-ups (panel **h**) in civilian Norwegian men (♂) and women (♀) at selection for military conscript service. The X-axis and percentile lines for panel **a**, **b**, **c**, **d**, **f** and **g** are truncated, i.e., extreme outliers are not displayed

Descriptive anthropometrical and physical fitness data are presented in Table 1. Cumulative relative frequencies with selected percentile values for BMI and the physical fitness variables are shown in Fig. 2. A more comprehensive set of percentile values for all variables are displayed in Online Resource 6. When adjusting the values in Table 1 for the possible selection bias, mean aerobic fitness and muscle strength were reduced by 3–8%, while the anthropometrical variables changed < 3% (Online Resource 7).

**Table 1:** Descriptive data on anthropometrics and physical fitness in Norwegian men and women at selection for military conscript service

Variable	*n*		Mean (SD)		Difference (men–women)		
	Men	Women	Men	Women	Mean diff. (95% CI)	*P*	ES
Height (cm)	102,689	51,952	180.8 (6.6)	167.6 (6.2)	+ 13.3 (13.2, 13.3)	< 0.001	2.05
Weight (kg)	102,337	51,803	75.8 (12.3)	64.5 (10.1)	+ 11.3 (11.2, 11.4)	< 0.001	0.97
Body mass index (kg·m^−2^)	102,325	51,798	23.1 (3.4)	22.9 (3.3)	+ 0.2 (0.2, 0.2)	< 0.001	0.06
Treadmill run time (min:sec)	94,500	46,027	11:45 (1:39)	9:45 (1:22)	+ 2:00 (1:59, 2:01)	< 0.001	1.28
Estimated VO_2peak_ (mL·kg^−1^·min^−1^)	94,500	46,027	52.9 (4.6)	42.7 (3.9)	+ 10.2 (10.2, 10.3)	< 0.001	2.32
Isometric chest press (kg)	62,078	30,094	127.0 (32.5)	74.7 (20.3)	+ 52.2 (51.8, 52.6)	< 0.001	1.80
Isometric leg press (kg)	61,349	29,754	312.9 (91.3)	217.0 (68.0)	+ 96.0 (94.8, 97.1)	< 0.001	1.14
Seated medicine ball throw (m)	21,503	13,352	3.15 (0.36)	2.20 (0.25)	+ 0.95 (0.94, 0.96)	< 0.001	2.96
Standing long jump (m)	21,481	13,281	2.25 (0.24)	1.79 (0.22)	+ 0.46 (0.46, 0.47)	< 0.001	2.03
Pull-ups (reps.)	18,847	11,927	7 (3–10)^a^	5 (2–10)^b^	N/A	< 0.001	1.93

Data are reported as means with standard deviations (*SD*), unless otherwise stated. Mean differences between genders are reported with 95% confidence intervals (*CI*), probability values (*P*) and effect sizes (*ES*)

^a^Values represent median (25–75 percentiles) number of repetitions of vertical pull-ups

^b^Values represent median (25–75 percentiles) number of repetitions of horizontal pull-ups

Among men, 3.7% were underweight, 19.1% were overweight and 4.6% were obese. The corresponding numbers were 3.9%, 17.7% and 3.7% among women. The frequency distribution of subjects by BMI categories was significantly different between men and women, *X*^2^ (3, *n* = 154,123) = 108, *P* < 0.001. Pairwise comparisons demonstrated significant differences in frequencies between men and women for overweight and obese (both *P* < 0.001), but not for underweight subjects (*P* = 0.07).

Men performed significantly better than women on all aerobic fitness and muscle strength measurements (Table 1). The largest effect size for the difference between men and women was demonstrated for the seated medicine ball throw (effect size 2.96). Effect sizes ≥ 1.1 were demonstrated for all physical fitness test variables. Women scored 59% of men’s performance in isometric chest press, while the corresponding figures were 69% for isometric leg press, 70% for seated medicine ball throw, 80% for standing long jump and 83% for treadmill run time (based on Table 1).

## Discussion

This study has presented reference data on physical fitness in young adult Norwegian men and women. The data can be used to evaluate current health and physical work capacity of young Norwegians, to act as norms for interpreting fitness test data in future national and international study samples and add to the body of literature investigating sex differences in physical fitness and performance. The discussion that follows are based on the unadjusted data, if not otherwise stated.

It is challenging to acquire true nationally representative data on objectively measured physical fitness. Many previous large-scale studies on aerobic capacity and muscle strength among youth and adults have experienced relatively low adherence rate (Danneskiold-Samsøe et al. 2009; Edvardsen et al. 2013; Loe et al. 2014). Thus, it is often hypothesized that physical fitness levels in previous reference studies are somewhat overreported (Hoffmann et al. 2019; Nes et al. 2013; Rapp et al. 2018; Aadland et al. 2017). The present study is not hampered by subjects declining to volunteer as conscript selection and service is obligatory. Still, the present data are somewhat skewed towards higher representativity of above-average fit subjects. The selection step one criteria have changed slightly over the years, but subjects who reported lower muscle strength or aerobic endurance compared to their peers of same sex and age were less likely to proceed to step two and thus to be included in the present study (Online Resource 7). Moreover, subjects who reported a BMI above 35 kg∙m^−2^ (obesity class II) or below 17 (clearly underweight) were usually excluded after step one. The same applied to subjects with medical restrictions (asthma, allergy, musculoskeletal disorders, etc.), low motivation for military service, low social score, drug use or poor school performance. Some of these criteria may correlate with physical fitness, and thereby indirectly eliminate a higher proportion of low-fit subjects. The supplementary study and analyses confirmed the selection bias, since the adjusted aerobic fitness and muscle strength figures were reduced by 3–8%, while anthropometrics changed < 3%, compared to the unadjusted means. Thus, for more correct general population estimates, the adjusted anthropometrical and physical fitness values presented in Online Resource 7 are most likely more accurate. However, these corrected means are built on assumptions and calculations, and should, therefore, be treated as estimations.

The maximal treadmill test used in the current study is especially designed for aerobic fitness testing at Norwegian conscript selection. Accordingly, we have no previous national or international data that can be used for direct comparisons of running performance. Yet, the presented estimated VO_2peak_ values can be compared to several previous reference studies on VO_2peak_ in Norwegian adolescence or young adults. Nes et al. (2013) demonstrated 12–15% higher mean VO_2peak_ (mL·kg^−1^·min^−1^) in 13- to 18-year-old boys and girls from the Nord-Trøndelag county, compared to the present data. Yet, another reference study from the same county have reported mean VO_2peak_ values in 20- to 29-year-old men and women to be very similar to the current data (Loe et al. 2014). Dyrstad et al. (2012) have published 3000 m run performance data for 17- to 18-year-old high school students from the city of Stavanger, Norway. If these run time data are converted into estimated VO_2peak_ from a nomogram (Bosquet et al. 2002), the mean values correspond very well with the present study. In addition, VO_2peak_ data on 15-year-old Norwegian pupils resemble the present study (Steene-Johannessen et al. 2009). Finally, Edvardsen et al. (2013) reported mean VO_2peak_ to be 6–8% lower in a sample of 20- to 29-year-old men and women from nine areas in Norway, compared to the present study. Overall, the current study seems to confirm previously reported mean VO_2peak_ values in healthy young adult Norwegian men and women. When compared to international reference data on VO_2peak_, the current values seem somewhat higher than what is typically reported in American and European young adult men and women (Hoffmann et al. 2019; Ingle et al. 2020; Rossi Neto et al. 2019; Wang et al. 2010; Wyss et al. 2019). Observed deviations against previous Norwegian and international data could of course be true differences among populations. Yet, differences may also be attributed to dissimilar test protocols (running vs. cycle ergometers), direct measurements vs. predicted values, differences in sampling (sample size, geographical catchment area, inclusion and exclusion criteria) and differences in age among study samples.

The isometric chest and leg press, seated medicine ball throw and pull-ups are also specially designed protocols for the Norwegian conscript selection and comparisons against previous reference data may be difficult. Opposingly, the standing long jump is a common field test for muscle power for which earlier reference data exist, particularly for children and young adults (Tomkinson et al. 2021). In a similar study as the present, Wyss et al. (2019) reported mean jump performance to be 2.31 m in 20-year-old Swiss males during conscript selection (no women included), which is 2.7% better than in the present study. On the other hand, mean jump distance in the present study was better than in 15-year-old boys and girls from the city of Kristiansand, Norway (Haugen et al. 2014), better than in 17-year-old school pupils from several European countries (Ortega et al. 2011) and marginally better than 19-year-old Finnish male and female conscripts (Santtila et al. 2018, 2019). Thus, lower body power seems relatively well developed in the reported subjects, compared to previous reference data.

The percentile distributions for BMI in males were remarkable similar to those reported in Swiss prospective conscripts (Wyss et al. 2019). However, the proportion of overweight or obese in the present study was somewhat higher than previously reported in 15-year-old Norwegian boys and girls (Haugen et al. 2014), but lower than in Norwegian men and women aged 20–29 years (Midthjell et al. 2013). Since anthropometric status usually changes significantly during adolescence and early adulthood (Bergh et al. 2016), such comparisons are hampered by age differences among the studies. It should also be repeated that BMI was an exclusion criterion during step one of the conscript selection process, although the exclusion thresholds were set wide (usually < 17 and > 35 kg·m^−2^). The wide thresholds, together with possible weight and height changes during the months or year(s) between step one and two examinations, are possible reasons why the current data still included a substantial number of underweight, overweight and obese subjects.

The magnitude of sex differences in fitness levels could be useful when evaluating test performances in sport, school and employment settings, or when establishing minimum requirements for physically demanding occupations. The present study demonstrated that the sex differences in physical performance varied substantially among different tests. The sex-related effect sizes were generally larger for upper body strength and power compared to lower-body measurements. This finding is concurrent with existing literature on differences in muscle strength between men and women (Bishop et al. 1987; Åstrand et al. 2003). There was also a tendency towards greater sex differences for power exercises compared to maximal isometric strength. The greatest difference between men and women was demonstrated for the seated medicine ball throw. Here, performance scores were similar at the 5th percentile for men and 95th percentile for women. The larger effect size for estimated VO_2peak_ compared to treadmill run time is probably due to the prediction equation used, which estimates women to have 4.6 mL·kg^−1^·min^−1^ lower VO_2peak_ for a similar run performance to men (Aandstad and Hageberg 2019).

Sex differences in muscle strength are primarily caused by larger muscle size in men than women, which again is caused by approximately 15 times greater circulating testosterone levels in men (Handelsman et al. 2018). Females’ lower running performance and aerobic power is also due to biological factors, such as lower blood hemoglobin levels, lower cardiac output (stroke volume) and higher fat mass (Åstrand et al. 2003). Yet, factors such as volume of training, motivation and competitiveness may also partly explain the observed sex differences (Åstrand et al. 2003). Data on physical activity levels of Norwegian children and adolescents over the last 2 decades indicate somewhat higher physical activity levels in boys than girls (Steene-Johannessen et al. 2021). Besides the biological factors, this lower physical activity level may have contributed to the sex differences in aerobic fitness and muscle strength observed in the present study.

Data from Norwegian conscript selection have previously been used to investigate secular changes in aerobic fitness in young adult Norwegian men. Dyrstad et al. (2005) demonstrated that estimated VO_2peak_ (mL·kg^−1^·min^−1^) was reduced by 8% from 1980 to 2002. Unfortunately, it is difficult to compare the Dyrstad et al. study with the present and evaluate whether a further decline in aerobic fitness has occurred. Earlier data were based on the submaximal Åstrand–Ryhming cycle ergometer test which underestimates VO_2peak_ compared to direct measurements carried out on cycle or treadmill ergometers (Dyrstad et al. 2005). The introduction of a two-step conscript selection system 10 years ago has also made comparisons to earlier data difficult, as the subjects who meet for physical examinations today are more narrowly selected than previously.

The present study is based on data collected over 2–8 years (depending on the test) and fitness levels may have changed over these years. A simple linear regression with fitness test performance as dependent variable, and year of examination as independent variable, indicates that mean (95% CI) treadmill run time improved by 3.0 (2.7, 3.2) seconds per year in men, and 3.3 (3.0, 3.6) seconds in women (data not shown). Similar, over the 5-year period with isometric strength testing, leg press performance increased by 1.0 (0.5, 1.6) kg and 2.0 (1.4, 2.5) kg per year in men and women, respectively, while chest press performance was reduced by 2.9 (2.7, 3.2) kg and 1.7 (1.6, 1.9) kg, respectively. The 2-year change in seated medicine ball throw, standing long jump and pull-ups were non-existing or negligible. Reasons for the observed fluctuations are not known but may be attributed to changes in selection step one criteria, wear or setup of equipment, minor changes in how the test leaders administered the tests or changes in physical fitness in the general young adult population.

The Norwegian Armed Forces emphasized validity, reliability, and practical aspects when implementing the chosen physical fitness tests at conscript selection (Kirknes et al. 2014). The treadmill test, as well as the medicine ball throw, standing long jump and pull-ups, may be recommended for use in other large-scale screenings and future reference studies of aerobic fitness and muscle strength and power in young and healthy individuals. These tests are relatively easy and quick to administer, do not require sophisticated equipment or lab facilities, and have demonstrated good reliability and validity (Aandstad 2015; Aandstad and Hageberg 2019; Aandstad and Kirknes 2018). In older subjects, alternative submaximal tests should be considered, due to increased health risk, injuries and discomfort associated with maximal testing.

### Study strengths and limitations

The most unique feature of the present study is its large sample size. Aerobic fitness is reported for approximately 140,000 subjects, and muscle strength for 127,000. This is much higher than in previous Norwegian reference studies of young adults and it is also higher than the majority of earlier international studies (Danneskiold-Samsøe et al. 2009; Rapp et al. 2018). Another unique aspect is that all geographical counties in Norway are represented in the material. Most previous reference studies are based on data from selected geographical cities or areas which may not be nationally representative (Nes et al. 2013; Ortega et al. 2011; Rossi Neto et al. 2019).

The present reference material includes data on aerobic fitness and muscle strength, which are both important components of physical fitness in relation to health, sport, and occupational performance (Hauschild et al. 2017; Liu et al. 2019; Ross et al. 2019; Åstrand et al. 2003). Aerobic fitness was tested with a maximal treadmill test which has demonstrated equally good reliability and validity to more established maximal treadmill tests like the Bruce or Balke protocol (Froelicher et al. 1975; Aadland et al. 2017). The inclusion of “whole-body” dynamic and isometric maximal strength and power measurements is also an advantage compared to many previous reference studies which focus solely on isometric strength tests, i.e., grip strength (Ahrenfeldt et al. 2019; Benfica et al. 2018).

A limitation of the present study is the under and over representativity of low and high fit subjects, respectively. The present study is, therefore, somewhat skewed towards higher mean values (including percentiles) compared to a fully representative sample for the general population. Yet, access to step one self-reported fitness data made it possible to take the selection bias into account and produce estimations of adjusted means. Such corrections are rare in previous reference studies.

Although the subjects were recruited from all 19 counties, information regarding county was not available at the individual level. It is, therefore, unknown if some counties were over or underrepresented in the study material. Moreover, the lack of individual county information precluded investigations of potential differences in physical fitness among regions and counties.

Another possible limitation is the use of an indirect aerobic fitness test. Direct measurements of VO_2peak_ is usually considered the gold standard measurement of integrated cardiopulmonary-muscle oxidative function (Poole and Jones 2017). However, it should be mentioned that running performance from an indirect test could perform equally well as a measure of health, sport and occupational performance (Noakes et al. 1990; Aadland et al. 2017). Reliability and validity of the measurements used in the present study are generally considered good, based on earlier method comparison studies (Aandstad 2015; Aandstad and Hageberg 2019; Aandstad and Kirknes 2018). However, no reliability data gathered at conscript selection were available (only during conscript service). The treadmill test, the isometric strength tests and the seated medicine ball throw are unique for Norwegian conscript selection, and previous reference material is, therefore, not directly available for comparison purposes.

Finally, it must be mentioned that some subjects may have underperformed on the tests, due to lack of familiarization with the test protocols or because of low motivation for military service. The latter was probably less relevant, as unmotivated subjects were usually screened out during step one of the selection process.

## Conclusions

The current study presents reference values for BMI, aerobic fitness and muscle strength and power in a large sample of young Norwegian men and women. Due to the selection procedure used by the Norwegian Armed Forces, it is estimated that the reported mean aerobic fitness and muscle strength values would be 3–8% lower in the general population. The present data can be used to evaluate current health and physical work capacity of young Norwegians, act as reference data against future population studies and contribute to quantifying differences in aerobic fitness and muscle strength between young men and women.

There is a general trend for declining participation rate in epidemiology research (Galea and Tracy 2007), and low participation rate and self-selection creates methodological challenges in many of today’s reference studies on physical fitness. Countries who practice obligatory military service may have a unique opportunity to gather more representative data on physical fitness in the young adult population. Thus, when planning descriptive studies on population fitness, it is worthwhile to consider research opportunities within the military system.

## Supplementary Information

Below is the link to the electronic supplementary material.

Supplementary file1 (PDF 224 KB)

Supplementary file2 (PDF 95 KB)

Supplementary file3 (PDF 91 KB)

## Data Availability

The data that support the findings of this study are available from the Norwegian Armed Forces HR and Conscription Centre, Hamar, Norway, but restrictions apply to the availability of these data, which were used under license for the current study, and so are not publicly available.

## Abbreviations

BMI: Body mass index
VO_2peak_: Peak oxygen uptake
