# Supplementary material for: Reference data on anthropometrics, aerobic fitness and muscle strength in young Norwegian men and women
Source: Eur J Appl Physiol. 2021 Aug 14;121(11):3189–200. doi: 10.1007/s00421-021-04784-4 (PMC8505311; doi:10.1007/s00421-021-04784-4)
Supplement: Supplementary file 1 — Supplementary file1 (PDF 224 KB) [file 421_2021_4784_MOESM1_ESM.pdf]

Reference data on anthropometrics, aerobic fitness and muscle strength in young Norwegian men and women

European Journal of Applied Physiology

Anders Aandstad, Norwegian Defence University College, Oslo, Norway

Corresponding author: Anders Aandstad, anaandstad@mil.no

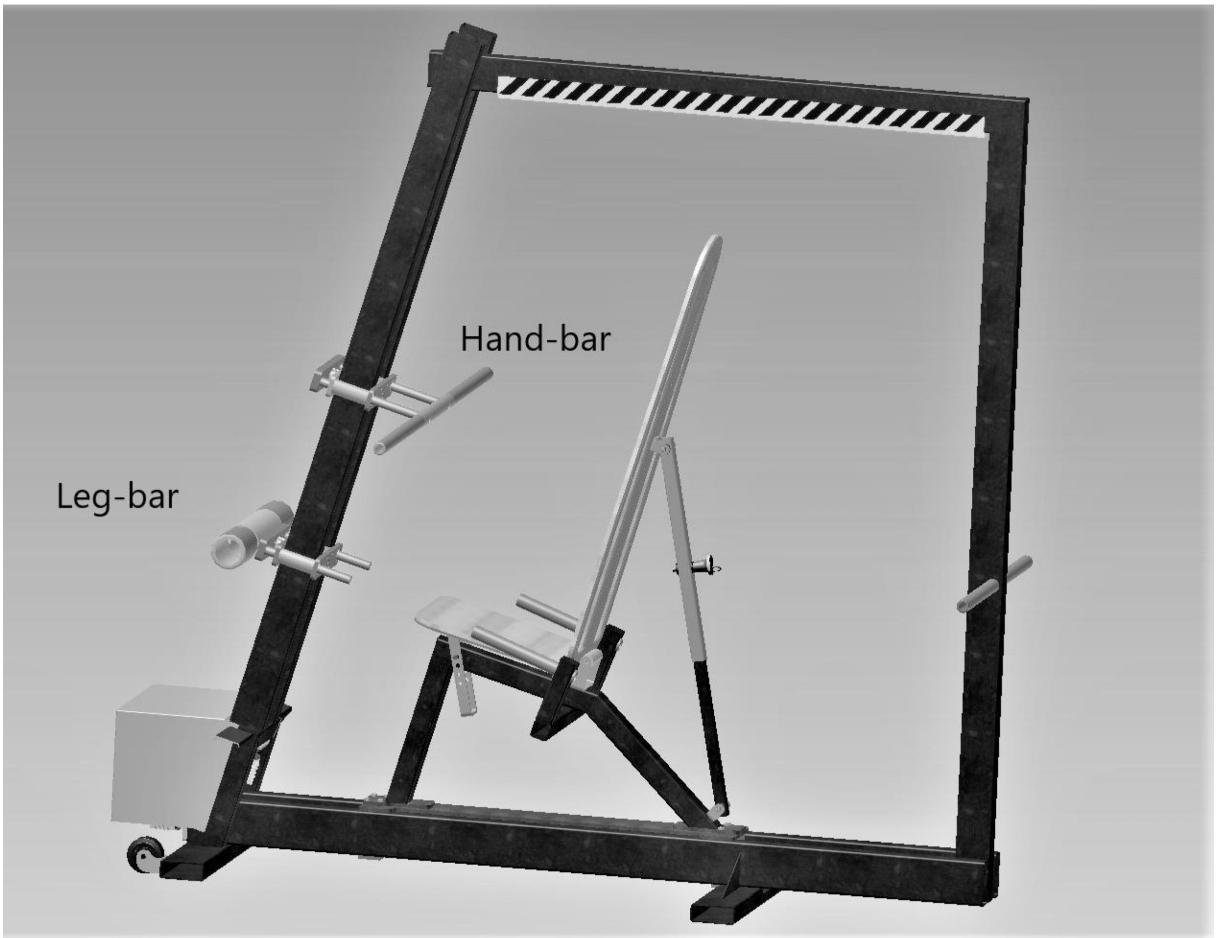

Online Resource 1. Illustration of the isometric chest and leg press apparatus.

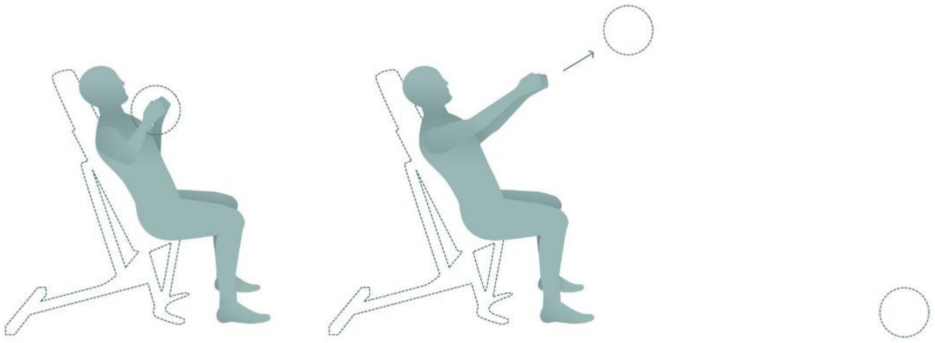

Online Resource 2. Illustration of the seated medicine ball throw test.

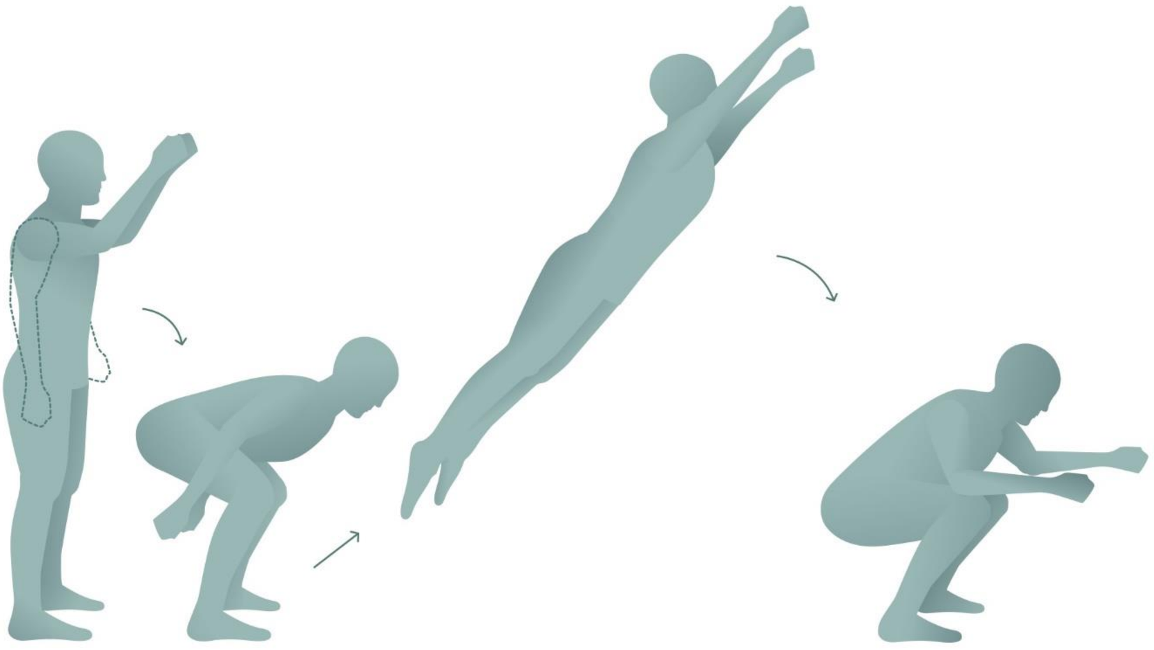

Online Resource 3. Illustration of the standing long jump test.

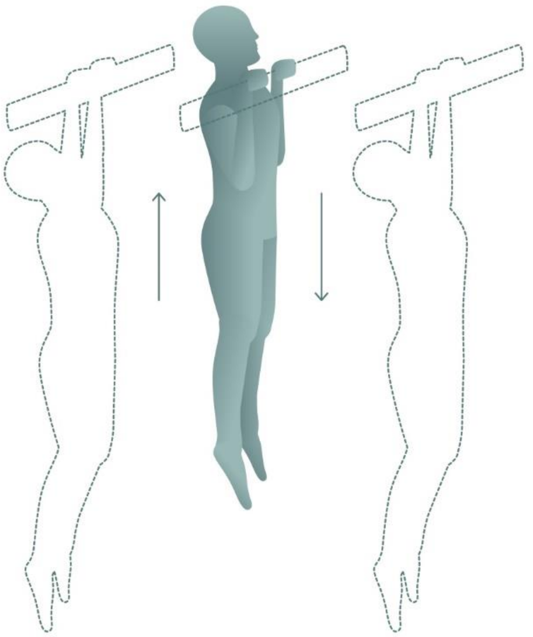

Online Resource 4. Illustration of the pull-ups vertical test.

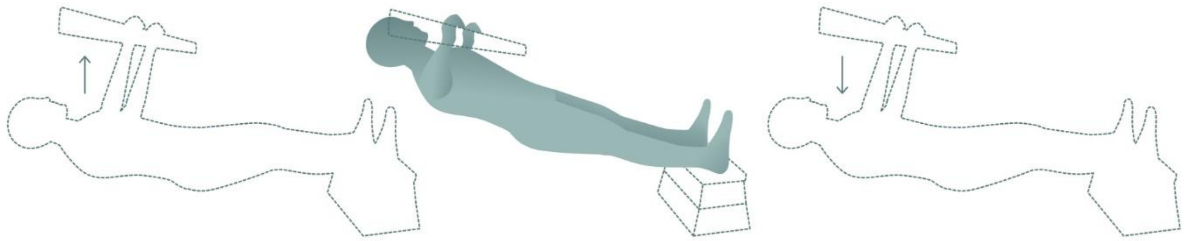

Online Resource 5. Illustration of the pull-ups horizontal test.
