# Supplementary material for: Reference data on anthropometrics, aerobic fitness and muscle strength in young Norwegian men and women
Source: Eur J Appl Physiol. 2021 Aug 14;121(11):3189–200. doi: 10.1007/s00421-021-04784-4 (PMC8505311; doi:10.1007/s00421-021-04784-4)
Supplement: Supplementary file 2 — Supplementary file2 (PDF 95 KB) [file 421_2021_4784_MOESM2_ESM.pdf]

Reference data on anthropometrics, aerobic fitness and muscle strength in young Norwegian men and women
European Journal of Applied Physiology
Anders Aandstad, Norwegian Defence University College, Oslo, Norway
Corresponding author: Anders Aandstad, anaandstad@mil.no

Online Resource 6. Percentiles for anthropometrics, aerobic fitness and muscle strength and power in Norwegian men and women at selection for military conscript service.

Sex	Variable	n	Percentiles										
			5th	10th	20th	30th	40th	50th	60th	70th	80th	90th	95th
Men	Height (m)	102,689	1.70	1.72	1.75	1.77	1.79	1.81	1.82	1.84	1.86	1.89	1.92
	Weight (kg)	102,337	59	62	66	69	71	74	77	80	84	91	98
	BMI ($kg \cdot m^{-2}$)	102,325	18.7	19.4	20.4	21.2	21.9	22.6	23.4	24.3	25.4	27.5	29.7
	Treadmill run time (min:sec)	94,500	09:00	09:45	10:17	11:00	11:15	12:00	12:01	12:39	13:02	14:00	14:26
	Est. VO_{2peak} ($mL \cdot kg^{-1} \cdot min^{-1}$)	94,500	45.2	47.3	48.8	50.8	51.5	53.6	53.7	55.5	56.6	59.3	60.5
	Isometric chest press (kg)	62,078	81	89	100	108	115	123	131	141	152	171	186
	Isometric leg press (kg)	61,349	183	204	233	256	278	301	324	353	393	455	500
	Seated medicine ball throw (m)	21,503	2.60	2.70	2.90	3.00	3.10	3.10	3.20	3.30	3.40	3.60	3.70
	Standing long jump (m)	21,481	1.85	1.95	2.05	2.15	2.20	2.25	2.30	2.40	2.45	2.50	2.60
	Pull-ups (reps.)	18,847	5-H	9-H	2-V	3-V	5-V	7-V	8-V	10-V	11-V	13-V	15-V
Women	Height (m)	51,952	1.57	1.60	1.62	1.64	1.66	1.68	1.69	1.71	1.73	1.75	1.78
	Weight (kg)	51,803	51	53	56	59	61	63	65	68	72	77	83
	BMI ($kg \cdot m^{-2}$)	51,798	18.7	19.4	20.3	21.1	21.8	22.4	23.2	24.0	25.1	27.1	29.1
	Treadmill run time (min:sec)	46,027	07:32	08:00	08:36	09:00	09:30	10:00	10:00	10:20	11:00	11:30	12:00
	Est. VO_{2peak} ($mL \cdot kg^{-1} \cdot min^{-1}$)	46,027	36.4	37.8	39.5	40.6	42.0	43.4	43.4	44.3	46.2	47.6	49.0
	Isometric chest press (kg)	30,094	47	51	58	63	67	72	77	83	90	102	112
	Isometric leg press (kg)	29,754	129	144	162	178	191	205	221	239	263	305	345
	Seated medicine ball throw (m)	13,352	1.80	1.90	2.00	2.10	2.10	2.20	2.25	2.30	2.40	2.50	2.60
	Standing long jump (m)	13,281	1.40	1.50	1.60	1.70	1.75	1.80	1.85	1.90	1.95	2.05	2.10
	Pull-ups (reps.)	11,927	0-H	0-H	1-H	3-H	4-H	5-H	7-H	9-H	12-H	2-V	4-V

BMI, body mass index; Est. VO_{2peak}, estimated peak oxygen uptake; H, horizontal; V, vertical
