# Supplementary material for: Reference data on anthropometrics, aerobic fitness and muscle strength in young Norwegian men and women
Source: Eur J Appl Physiol. 2021 Aug 14;121(11):3189–200. doi: 10.1007/s00421-021-04784-4 (PMC8505311; doi:10.1007/s00421-021-04784-4)
Supplement: Supplementary file 3 — Supplementary file3 (PDF 91 KB) [file 421_2021_4784_MOESM3_ESM.pdf]

Reference data on anthropometrics, aerobic fitness and muscle strength in young Norwegian men and women
European Journal of Applied Physiology
Anders Aandstad, Norwegian Defence University College, Oslo, Norway
Corresponding author: Anders Aandstad, anaandstad@mil.no

Online Resource 7. Descriptive data for anthropometrics, aerobic fitness and muscle strength and power in Norwegian men and women at selection for military conscript service. The sample mean represents true mean (SD) for data gathered at selection step two. The adjusted mean represents a general population estimate, based on supplementary analyses (including weighted means). The difference (Δ) in percent is calculated as ((adjusted mean - sample mean)/sample mean) x 100.

	Men			Women		
Variable	Sample mean	Adjusted mean	Δ %	Sample mean	Adjusted mean	Δ %
Height (cm)	180.8 (6.6)	180.1	-0.4	167.6 (6.2)	166.9	-0.4
Weight (kg)	75.8 (12.3)	77.2	+1.8	64.5 (10.1)	65.3	+1.2
Body mass index ($kg \cdot m^{-2}$)	23.1 (3.4)	23.7	+2.6	22.9 (3.3)	23.4	+2.2
Treadmill run time (min:sec)	11:45 (1:39)	11:11	-4.8	9:45 (1:22)	09:11	-5.8
Estimated VO_{2peak} ($mL \cdot kg^{-1} \cdot min^{-1}$)	52.9 (4.6)	51.3	-3.0	42.7 (3.9)	41.0	-3.9
Isometric chest press (kg)	127.0 (32.5)	122.3	-3.7	74.7 (20.3)	71.3	-4.6
Isometric leg press (kg)	312.9 (91.3)	302.3	-3.4	217.0 (68.0)	208.3	-4.0
Seated medicine ball throw (m)	3.15 (0.36)	3.06	-3.0	2.20 (0.25)	2.05	-6.7
Standing long jump (m)	2.25 (0.24)	2.15	-4.6	1.79 (0.22)	1.65	-8.0
Pull-ups (reps.)	7 (3–10)†	N/A	N/A	5 (2–10)‡	N/A	N/A

† Values represent median (25–75 percentiles) number of repetitions of vertical pull-ups.
‡ Values represent median (25–75 percentiles) number of repetitions of horizontal pull-ups.
N/A, not available

Description of supplementary study and calculation of adjusted means

Subjects
All subjects born year 1996 (31,350 men and 29,267 women) and year 2000 (29,736 men and 28,377 women) who had completed the self-reported questionnaire during step one of the conscript selection were included in the analyses. This comprised ≥95% of the entire nationwide age cohort of 17 year old men and women born 1996 and 2000.

Measurements
Both cohorts reported their height and weight to the nearest cm and kg, respectively. Subjects born 1996 indicated their self-perceived endurance and muscle strength level on a five-point Likert-scale (“much worse”, “worse”, “similar”, “better”, “much better” – compared to peers of same age and gender). Those born 2000 carried out physical fitness tests at home and reported their 3,000 m run time, as well as push-ups, standing long jump and pull-ups performance within pre-defined categories (e.g., 0, 1-4, 5-9 push-ups, etc.). Approximately one third of the subjects passed step one and were selected to step two. Subjects born in 1996 typically met at conscript selection step two in 2014/15 and performed the maximal treadmill test and the isometric chest and leg press tests. Subjects born 2000 typically met at conscript selection step two in 2018/19 and performed the maximal treadmill test, seated medicine ball throw, standing long jump and pull-ups.

Analyses

Adjusted means for the anthropometrical variables (height, weight, and BMI) were calculated as follows:
The percent difference of self-reported (step one) anthropometrics for the entire sample and those selected to step two were calculated. As an example, mean self-reported weight for all men born 1996 and 2000 was 74.6 kg, while the corresponding weight for those being selected to step two was 73.3 kg. Thus, mean weight was 1.3 kg (1.8%) higher among subjects in the entire step one sample compared to those who were selected to participate in step two. Accordingly, men's mean weight measured at selection step two were adjusted by +1.8%, from 75.8 to 77.2 kg (see Table above). Similar adjustments were made for men and women (separately) for all anthropometrical variables.

Adjusted means for all aerobic fitness and muscle strength variables were calculated as follows:
A higher percent of those indicating good muscle strength and aerobic fitness at step one was selected to participate in step two, compared to those indicating lower strength and aerobic fitness. Weighted means were therefore calculated to account for this bias. An example is given for treadmill run time: At selection step one, 1,408 out of 31,350 men (4.5%) indicated that their cardiorespiratory endurance was "much worse" compared to peers of the same age and sex. Only 17 of those 1,408 (1.2%) were selected to step two. Mean treadmill run time for these 17 were 09:12 min:sec at step two. On the other hand, 2,221 out of 31,350 men (7.1%) indicated at step one that their cardiorespiratory endurance was "much better" compared to peers of the same age and sex. Of these 2,221 men, 1,484 (66.8%) were selected to step two, where their mean treadmill run time was 13:31 min:sec. For the adjusted analyses, the mean values for treadmill run were given weight according to the step one percentage. Accordingly, the 17 men who produced a mean treadmill run time of 09:12 min:sec were given a 4.5% weight when calculating the adjusted mean values. Similar, the 1,484 men who produced a treadmill time of 13:31 min:sec were given a 7.1% weight to the adjusted mean values. Similar calculations were performed for those who indicated that their cardiorespiratory endurance was "worse" (23.3%), "similar" (38.8%) and "better" (26.3%). The unadjusted mean value for treadmill run time for men born in 1996 were 11:47 min:sec, while the adjusted mean was 11:13 min:sec (i.e., 4.8% lower). Accordingly, the sample mean value in the table above (11:45 min:sec) were reduced by 4.8% to produce the adjusted mean value of 11:11 min:sec.

A similar approach was used for both genders and for all cardiorespiratory endurance and muscle strength variables.

Discussion

The abovementioned adjustments are based on at least two assumptions. The first assumption is that the difference between sample mean and adjusted mean for one age cohort (e.g. those born in 1996) are representative for all age cohorts included in the main study. This assumption is probably close to true since the step one selection procedure has changed little within the years 2011 to 2017 and 2017 to 2019.
Another assumption is that subjects in a particular self-reported category of physical fitness who passed selection step one (e.g. the 17 men reporting much worse aerobic fitness compared to their peers), are representative (pertaining treadmill run performance) for the subjects who did not pass step one (i.e., the remaining 1,391 men who reported that their aerobic fitness was much worse compared to their peers). It is unknown how well this assumption holds, but there are reasons to believe it is also close to true.
Since the adjusted mean values are based on certain assumptions, they should be viewed as estimations.
